# Genome-Wide Identification of MicroRNAs and Their Targets in the Leaves and Fruits of *Eucommia ulmoides* Using High-Throughput Sequencing

**DOI:** 10.3389/fpls.2016.01632

**Published:** 2016-11-08

**Authors:** Lin Wang, Hongyan Du, Ta-na Wuyun

**Affiliations:** ^1^Non-timber Forest Research and Development Center, Chinese Academy of ForestryZhengzhou, China; ^2^The Eucommia Engineering Research Center of State Forestry AdministrationZhengzhou, China

**Keywords:** *Eucommia ulmoides*, trans-1, 4-polyisoprene, miRNAs, high-throughput sequencing, target genes

## Abstract

MicroRNAs (miRNAs), a group of endogenous small non-coding RNAs, play important roles in plant growth, development, and stress response processes. *Eucommia ulmoides* Oliver (hardy rubber tree) is one of the few woody plants capable of producing trans-1, 4-polyisoprene (TPI), also known as Eu-rubber, which has been utilized as an industrial raw material and is extensively cultivated in China. However, the mechanism of TPI biosynthesis has not been identified in *E. ulmoides*. To characterize small RNAs and their targets with potential biological roles involved in the TPI biosynthesis in *E. ulmoides*, in the present study, eight small RNA libraries were constructed and sequenced from young and mature leaves and fruits of *E. ulmoides*. Further analysis identified 34 conserved miRNAs belonging to 20 families (two unclassified families), and 115 novel miRNAs seemed to be specific to *E. ulmoides*. Among these miRNAs, fourteen conserved miRNAs and 49 novel miRNAs were significantly differentially expressed and identified as Eu-rubber accumulation related miRNAs. Based on the *E. ulmoides* genomic data, 202 and 306 potential target genes were predicted for 33 conserved and 92 novel miRNAs, respectively; the predicted targets are mostly transcription factors and functional genes, which were enriched in metabolic pathways and biosynthesis of secondary metabolites. Noticeably, based on the expression patterns of miRNAs and their target genes in combination with the Eu-rubber accumulation, the negative correlation of expression of six miRNAs (Eu-miR14, Eu-miR91, miR162a, miR166a, miR172c, and miR396a) and their predicted targets serving as potential regulators in Eu-rubber accumulation. This study is the first to detect conserved and novel miRNAs and their potential targets in *E. ulmoides* and identify several candidate genes potentially controlling rubber accumulation, and thus provide molecular evidence for understanding the roles of miRNAs in regulating the TPI biosynthesis in *E. ulmoides*.

## Introduction

*Eucommia ulmoides* Oliver is a deciduous tree and a species of the only genus in the family Eucommiaceae (euasterids I: order Garryales) (Call and Dilcher, 1997). It has been used in traditional Chinese medicine and extensively cultivated across 27 provinces in China. This species is also one of the best-known trans-1,4-polyisoprene (TPI) producers (Takeno et al., 2008; Du et al., 2015). TPI, also known as *Eucommia* rubber (Eu-rubber), is extracted from leaves, fruits, roots, and bark of *Eucommia*. Its content varies in different tissues and at different plant developmental stages, with the highest content found in fruits (Du, 2003; Yoshihisa et al., 2009). Eu-rubber, which shows less flexibility and poorer low-temperature thermoplasticity than cis-polyisoprene rubber, has attracted considerable attention as a raw industrial material for various commercial uses (Weiss, 1891; Jiang et al., 2006; Chen et al., 2008). At present, rubber tree (*Hevea brasiliensis*) is the most widely cultivated species for commercial production of natural rubber. However, as a typical tropical rainforest plant, this species is restricted to the tropics where it has already reached the limit of production capacity (Webster and Baulkwill, 1989; Suzuki et al., 2012; Wang Y. et al., 2013; Wang, 2014). Therefore, the shortage of natural rubber and the growing worldwide demand for the same necessitate an urgent development of Eu-rubber industry, which will meet the demand by increasing the economic yield and improving the content of Eu-rubber.

Previous studies have focused primarily on pharmacological and morphological aspects of *E. ulmoides*, and only limited genomic and molecular information is available. Furthermore, the biosynthetic enzymes involved in the synthesis and regulation of Eu-rubber are not well studied. In plants, the precursors of polyisoprene are derived from two distinct pathways: the mevalonate (MVA) pathway in cytoplasm and the methylerythritol phosphate (MEP) pathway in plastids (Yin et al., 2011). High-molecular-weight polyisoprene is synthesized by successive condensation of the precursors via a specific prenyltransferase (Suzuki et al., 2012). Although most MEP and MVA pathway genes have been cloned and analyzed, their function in Eu-rubber accumulation is not fully understood.

Small RNAs (sRNAs) contain endogenous small interfering RNAs and microRNAs (miRNAs) that are 21–24 nucleotides (nt) long (Jones-Rhoades et al., 2006). Small interfering RNAs are processed from perfectly double-stranded RNA, and miRNAs are derived from single-stranded RNA transcripts that form imperfectly double-stranded stem loop precursor structures (Llave et al., 2002a; Khraiwesh et al., 2010; Hao et al., 2012; Szittya and Burgyan, 2013). miRNAs are the most typical plant sRNAs that negatively regulate expression of target genes by repressing gene translation or cleaving target mRNAs. miRNAs play a vital role in various biological and metabolic processes of plant growth and development, such as signal transduction and responses to biotic or abiotic stresses (Bartel, 2004; Jones-Rhoades and Bartel, 2004; Jones-Rhoades et al., 2006; Mallory and Vaucheret, 2006; Sunkar et al., 2006; Voinnet, 2009; Wu et al., 2010). Furthermore, many plant miRNAs show tissue- or stage-specific expression patterns; for example, miR159, miR167, and miR172 are expressed between male and female flowers in asparagus, and miR156g and miR169t are stage-specific in *Lycium barbarum* fruits (Zeng et al., 2015; Chen et al., 2016). The expression patterns of miR156f-3p, miR157a-3p, miR5021, miR5163, miR5293, and novel_miR_27 are different in *Panax notoginseng* roots, stems, and leaves at different developmental stages (Wei et al., 2015). miRNAs also participate in regulating terpenoid accumulation through regulating their target genes, and in *Arabidopsis thaliana* and tomato, flavonoid biosynthesis is affected by the expression levels of miRNAs (Davuluri et al., 2005; Gou et al., 2011; Ng et al., 2011); miR6435, miR5021, and miR1134 are specifically expressed in glandular trichomes of *Xanthium strumarium* and play a role in the regulation of xanthanolide biosynthesis (Fan et al., 2015); and miR156, miR828, miR858, and miR5072 regulate anthocyanin accumulation in apple peels (Qu et al., 2016). The expression of miR159b regulates latex production in *H. brasiliensis* (Gébelin et al., 2013), and the negative regulation of hbr-miR172 results in a dramatic increase in latex yield of the rubber tree (Pramoolkit et al., 2014). Therefore, it is important to identify miRNAs and their target genes, which is essential to understanding miRNAs-mediated gene regulation of Eu-rubber biosynthesis.

miRNAs have been thoroughly studied in many species. Although *E. ulmoides* has significant medicinal and economical value, miRNAs are yet to be identified in this species. Many studies have confirmed that both conserved and species-specific miRNAs are very important in different biological processes in plants. Therefore, the aim of this study was to identify the conserved and novel miRNAs and their potential target genes in *E. ulmoides* and explore their interactions. To achieve this, we sequenced sRNA from leaves and fruits at different developmental stages by using the Illumina platform, analyzed gene expression profiles, and investigated the functions of their targets. Quantitative real-time PCR (qPCR) was adopted to evaluate the expression levels of miRNAs and their target genes and identify candidate genes involved in Eu-rubber accumulation. Our results provide valuable information about miRNAs involved in rubber biosynthesis in *E. ulmoides*.

## Materials and methods

### Plant materials

Leaves and fruits of *E. ulmoides* “Huazhong No. 6” were collected at Non-timber Research and Development Center of CAF (113°41′37″E, 34°46′23″N, Zhengzhou, Henan, China) on May 3 (at young stage) and July 16 (at mature stage) in 2015. The samples were labeled YL (young leaf, 0.26%, the rate of Eu-rubber accumulation), ML (mature leaf, 0.29%), YF (young fruits, 1.67%), and MF (mature fruits, 0.07%). The leaves and fruits were collected from three randomly selected individuals and pooled together in a single biological sample; two biological replicates were prepared for high-throughput sequencing. All samples were immediately frozen in liquid nitrogen and stored at −80°C for further analyses.

### Total RNA isolation, sRNA library construction, and sequencing

Total RNAs were extracted from YL, ML, YF, and MF using a TRIzol reagent (Invitrogen, Carlsbad, CA, USA) according to the manufacturer's instructions. RNA quality was monitored on 1% agarose gels. RNA concentration was measured with a Qubit 2.0 Fluorometer (Life Technologies, Carlsbad, CA, USA), and the integrity was assessed by an Agilent Bioanalyzer 2100 (Agilent Technologies, Santa Clara, CA, USA). The purified RNAs were used to construct sRNA libraries and were sequenced on an Illumina HiSeq 2500 platform at Novogene Company, Beijing, China.

### Small RNA analysis and miRNAs prediction

Raw sequence data were obtained via the RNA high-throughput sequencing process and screened to remove contaminated reads, sequences containing “adapters,” those without insert tags, and reads with poly-A tails. In addition, Q20, Q30, and GC content of the raw data was calculated. The sequences 18–30 nt long were used for further analysis. Clean reads of sRNA tags were mapped onto the *E. ulmoides* genome database (unpublished) without mismatch to analyze their expression and distribution using Bowtie (Langmead et al., 2009). The rRNA, tRNA, snRNA, and snoRNA were annotated by aligning them against the Rfam database (Rfam:http://www.sanger.ac.uk/software/Rfam) and the NCBI GenBank databases (GenBank:http://www.ncbi.nlm.nih.gov/blast/Blast.cgi). Natural antisense transcripts and trans-acting small interfering RNAs were identified by using the PlantNATsDB (http://bis.zju.edu.cn/pnatdb/) and UEA sRNA tools, respectively (Moxon et al., 2008; Chen et al., 2011). The repeat sequences were annotated by aligning them to the Repbase database by RepeatMasker (Bao et al., 2015; Smit et al., 2013-2015). The mapped sRNA tags were used to search for conserved miRNA. miRBase version 21(ftp://mirbase.org/pub/mirbase/21/) was used as a reference, and modified software miRDeep2 and srna-tools-cli were used to obtain the potential miRNA sequences and draw the secondary structures (Friedländer et al., 2011). The available software miREvo and miRDeep2 were integrated to predict novel miRNA by exploring the secondary structure, the Dicer cleavage site, and the minimum free energy of the sRNA tags unannotated in the former steps (Wen et al., 2010; Friedländer et al., 2011). To explore the occurrence of miRNA families, we used the conserved miRNAs in miFam.dat (http://www.mirbase.org/ftp.shtml), and the novel miRNA precursors were aligned to Rfam (http://www.rfam.sanger.ac.uk/search/).

### Differential expression analysis of miRNAs

To calculate significant differences in expression patterns in miRNAs, the expressions of miRNAs were estimated using transcripts per million (TPM) according to the normalization formula: Normalized expression = Mapped read count/Total reads × 1,000,000 (Zhou et al., 2010). Differential expression analysis of the two groups of biological replicates was performed for the samples using DESeq R package (1.8.3) (Anders and Huber, 2010). The *P*-values were adjusted using the Benjamin & Hochberg method, and corrected *P*-value of 0.05 was set as a threshold for significant differential expression by default (Love et al., 2014).

### Target gene prediction and functional annotation

The target genes of miRNAs were predicted with the Web-based psRNATarget program (http://plantgrn.noble.org/psRNATarget/) (Dai and Zhao, 2011). The gene ontology (GO)-biological process and Kyoto Encyclopedia of Genes and Genomes (KEGG) pathway terms enriched in the target gene candidates were determined using GOseq and KOBAS software, respectively (Mao et al., 2005; Young et al., 2010).

### Quantitative real-time PCR (qPCR) analysis

To obtain candidate genes that were potentially controlling rubber accumulation, the expression level of several miRNAs and their potential targets were selected for qPCR. Total RNA was extracted from young and mature leaves or fruits by using a TRIzol reagent (Invitrogen, USA). The extracted total RNA was reverse-transcribed into cDNA using an AMV First Strand cDNA Synthesis Kit (Sangon, Shanghai, China) to detect the expression levels of miRNAs and their target genes. For miRNA expression, stem-loop qPCR primers were designed as previously described (Varkonyi-Gasic and Hellens, 2011), and the primers for target genes were designed by using Primer 5.0 (Premier Biosoft International, Palo Alto, CA, USA). Actin α was used as an internal reference gene for normalization; the primers used for qPCR were listed in Table S1. qPCR was performed using an ABI StepOnePlus system (Applied Biosystems, Foster City, CA, USA). Each PCR was performed in a reaction volume of 20 μL containing 10 μL of SybrGreen qPCR Master Mix (2 ×), 0.4 μL of forward primer, 0.4 μL of reverse primer, 2 μL of reverse-transcribed cDNA, and 7.2 μL of ddH2O. The PCR conditions were as follows: 3 min at 95°C, 45 cycles of 95°C for 7 s, 57°C for 10 s, and 72°C for 15 s, and melt curve analysis from 60 to 95°C. All reactions were performed in triplicate for each sample, and the expression levels were calculated with the 2^−ΔΔCt^ method (Livak and Schmittgen, 2001). All the data were subjected to an analysis of variance and the results were presented as the mean ± SD. The *P*-value was considered to be statistically significant if <0.05 or <0.01.

## Results

### Sequence analysis of eight sRNA libraries

**Figure 1:**
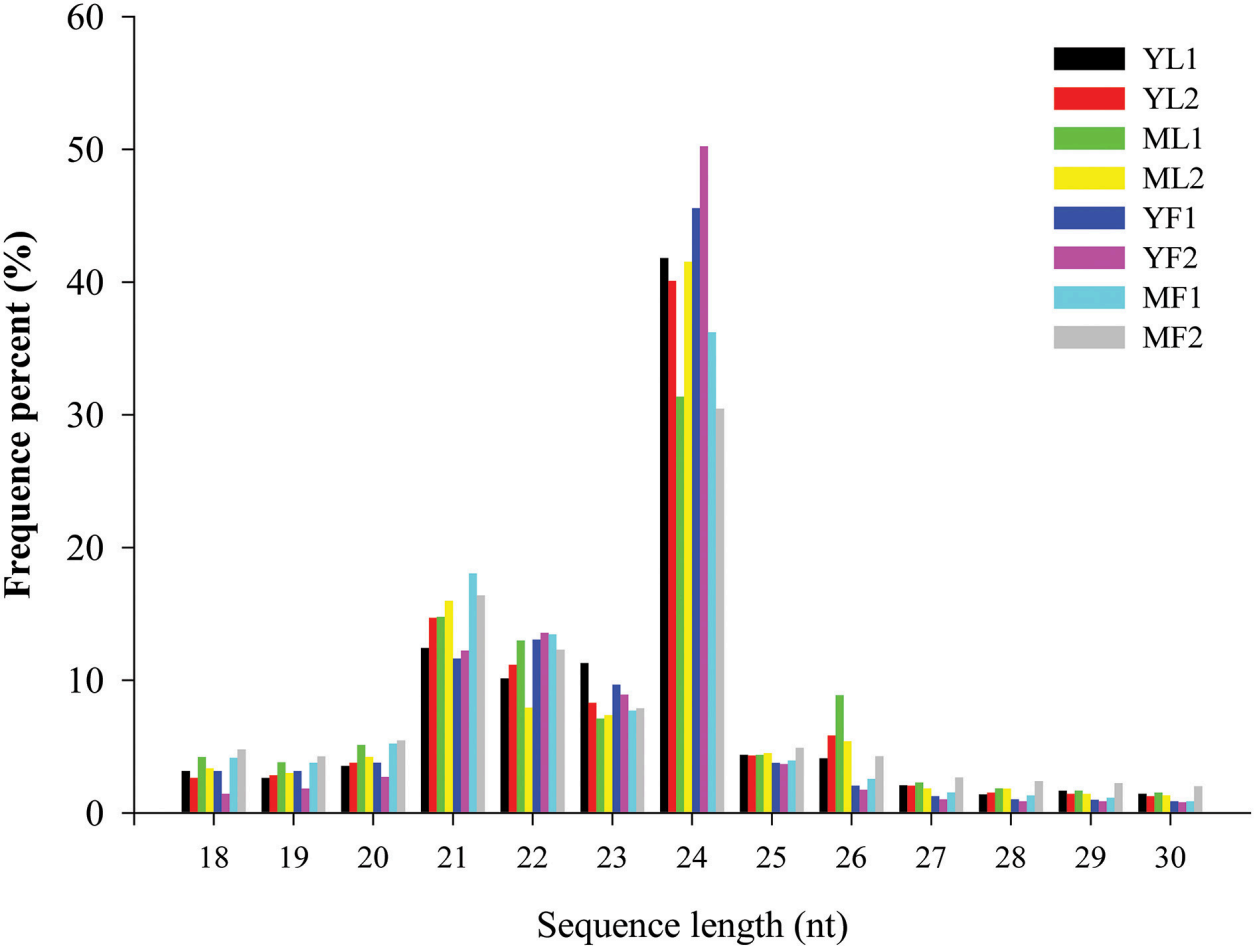
**Length distribution and abundance of small RNAs in eight libraries of *Eucommia ulmoides***. X-axis, length of sRNA distribution; Y-axis, corresponding percentage of raw reads.

To identify the miRNAs in *E. ulmoides*, the libraries from young leaves, mature leaves, young fruits, and mature fruits were generated and sequenced via the Illumina HiSeq 2500 platform. By using two biological replicates of the leaf and fruit tissues collected at two different developmental stages, eight cDNA libraries (YL1, YL2, ML1, ML2, YF1, YF2, MF1, and MF2) were created from *E. ulmoides*, yielding approximately 98 million raw reads. After removing the low-quality sequences and adapter sequences, a total of 9,885,853 (YL1), 12,288,265 (YL2), 11,482,801 (ML1), 11,336,124 (ML2), 10,371,302 (YF1), 12,588,040 (YF2), 14,889,857 (MF1), and 12,177,627 (MF2) clean reads 18–30 nt long were generated (Table 1). To verify the quality of the libraries, we inspected the error rate and GC content of the sequencing results (Table S2). Furthermore, 60.86–72.32% of high-quality sRNA reads from each of the eight libraries were mapped to the *E. ulmoides* genome, suggesting no significant sequencing bias in these eight libraries. The distribution pattern of sRNA was similar among the eight libraries. The size distribution of sRNAs was uneven, with the majority (>75%) of sRNAs having 20–24 nt and those with 24 nt being the most abundant class. The second most abundant class comprised sRNAs 21 nt long, and the proportion of the sRNAs length dynamically changed in different tissues and at different developmental stages (Figure 1). Various types of non-coding RNAs, which comprised conserved miRNAs, putative novel miRNAs, rRNAs, tRNAs, snRNAs, snoRNAs, repeat-associated RNAs, natural antisense transcripts, trans-acting small interfering RNAs, and unannotated fragments, were annotated (Table S3).

**Table 1:** **Statistical summary of the data generated by high-throughput sequencing in *Eucommia ulmoides***.

**Sample**	**Raw reads**	**Low quality reads**	***N*% > 10%**	**5′ adapter contaminants**	**3′ adapter null**	**Poly (A/T/G/C)**	**Clean reads**	**Mapped**
YL1	10,209,939	5,200	89	9,708	277,060	32,029	9,885,853	4,755,375
	100.00%	0.05%	0.00%	0.10%	2.71%	0.31%	96.83%	68.32%
YL2	12,697,754	1,196	630	10,474	355,960	41,229	12,288,265	6,227,619
	100.00%	0.01%	0.00%	0.08%	2.80%	0.32%	96.78%	62.00%
ML1	11,860,292	1,313	1,213	9,171	334,459	31,335	11,482,801	5,855,866
	100.00%	0.01%	0.01%	0.08%	2.82%	0.26%	96.82%	63.27%
ML2	11,809,421	1,787	1,177	6,956	415,101	48,276	11,336,124	6,634,384
	100.00%	0.02%	0.01%	0.06%	3.51%	0.41%	95.99%	68.71%
YF1	10,666,582	6,268	113	12,186	223,547	53,166	10,371,302	5,381,106
	100.00%	0.06%	0.00%	0.11%	2.10%	0.50%	97.23%	60.86%
YF2	13,061,860	3,529	686	15,011	389,222	65,372	12,588,040	8,346,534
	100.00%	0.03%	0.01%	0.11%	2.98%	0.50%	96.37%	72.32%
MF1	15,314,563	1,546	1,538	11,316	366,225	44,081	14,889,857	6,100,081
	100.00%	0.01%	0.01%	0.07%	2.39%	0.29%	97.23%	62.42%
MF2	12,496,929	1,369	1,239	8,474	271,635	36,585	12,177,627	7,830,332
	100.00%	0.01%	0.01%	0.07%	2.17%	0.29%	97.44%	60.97%

*N%, undetectable gaps at base calling*.

### Identification of conserved miRNAs

**Figure 2:**
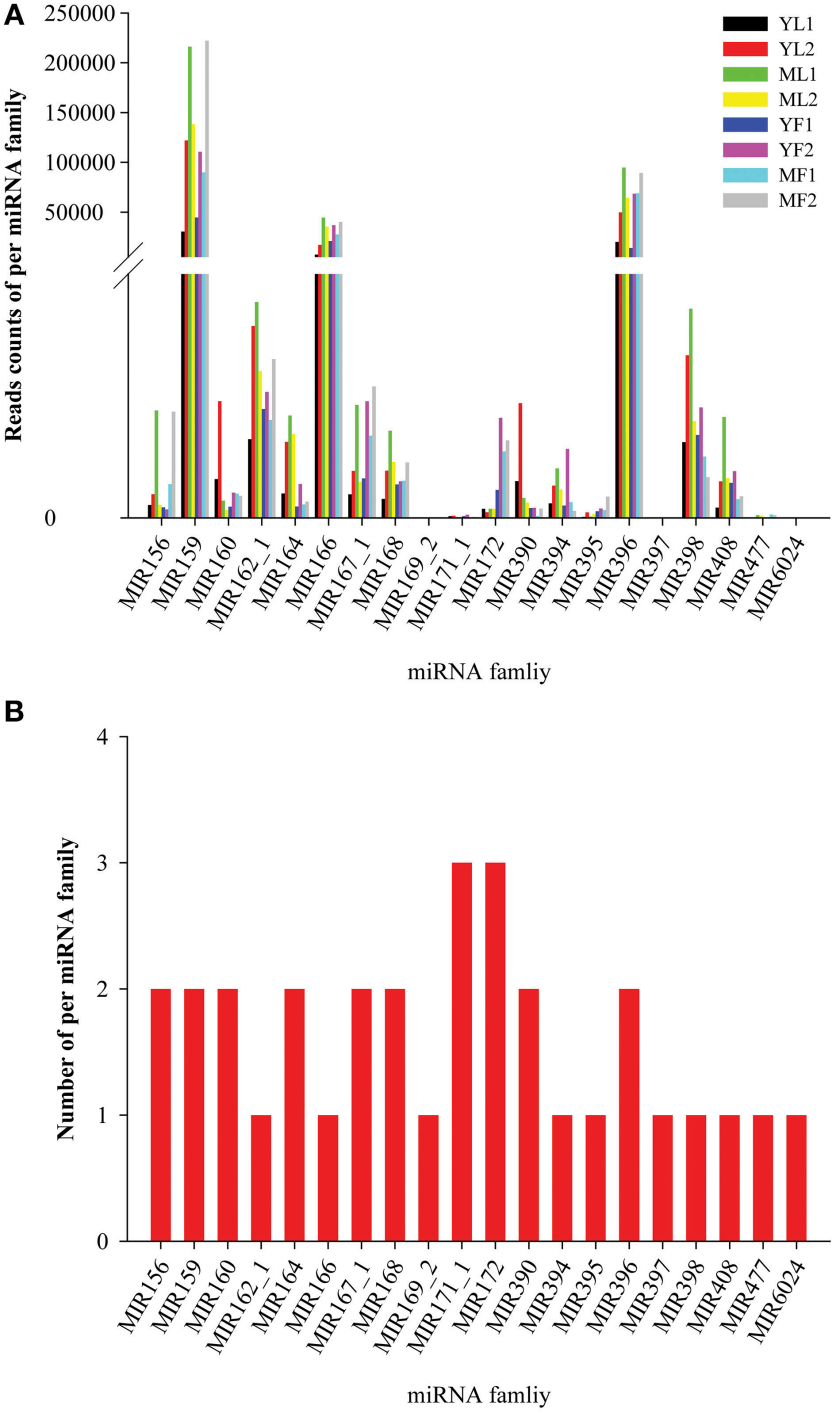
**Abundance (A)** and distribution of the member numbers **(B)** in each conserved miRNA family in *Eucommia ulmoides*.

Conserved miRNA families are found in many plant species, wherein they are crucial for plant development. To identify the conserved miRNAs, the eight sRNA libraries were subjected to BLASTN search for conserved mature miRNAs and precursors in miRbase database. A total of 34 conserved miRNAs (belonging to 20 miRNA families and two unclassified families) that were orthologs of conserved miRNAs from other plant species were found in the eight libraries of *E. ulmoides* (Table S4). The length of the conserved miRNAs varied from 20 to 22 nt, with the majority (79.41%) having 21 nt. Interestingly, the abundance and the number of miRNA in each miRNA family were considerably different. Among these families, the most abundant was MIR159 (read counts > 10,000), whereas MIR397 and MIR6024 had the lowest abundance (read counts <10). The expression levels of the same miRNA family in each library varied significantly; for instance, the read numbers of MIR166 were 44,586 in MF1, whereas those in YF1 were only 7,381 (Figure 2A). Similarly, the expression levels of different members in each miRNA family were also different; in the miR164 family, miR164a was highly expressed in both leaf and fruit tissues, whereas the expression of only miR164c was low in fruits. In addition, the number of these miRNAs varied widely between families: 11 families had a single member, 8 families had 2 members, and 2 families possessed 3 members (Figure 2B). The conserved miRNA precursors with lengths ranging from 99 to 273 nt were identified. Nucleotide sequence analysis of these conserved miRNAs revealed a strong preference for adenosine (A) at the 10th position and for uridine (U) at the 1st position. We also analyzed the number of reads for conserved miRNAs in the eight libraries. As expected, miRNAs had a very broad range of expression, from millions of sequence reads to fewer than 10 sequence reads, and some miRNAs showed clear tissue- or stage-specific expression. The most abundant miRNA was miR159 (up to 53.55% of the total sequence reads). The expression levels of miR156a, miR159, miR396, and miR477 in mature tissues were higher than those in young tissues, whereas the expression of miR162a and miR171c was greater in young tissues. In addition, miR167a, miR172a/172c/172j, and miR395a were the most abundant in the leaf libraries, whereas miR164c, miR6024, and miR1446 were fruit-specific and miR169 was found only in mature leaves, although at a low expression level (<10).

### Identification of novel miRNAs

To search for potentially novel miRNAs in *E. ulmoides*, miREvo and miRDeep 2 software were used to explore the stem-loop hairpin secondary structure and the dicer cleavage site and to measure the minimum free energy of the novel miRNA sequences. A total of 115 novel hairpin miRNAs candidates between 18 and 24 nt long were identified; these miRNA candidates are likely to be new miRNAs or new members of conserved miRNA families in *E. ulmoides* (Table S5). The length of the precursors in novel miRNAs ranged from 51 to 296 nt, with an average of 133 nt; this was consistent with the typical length distribution of mature miRNAs. The nucleotide bias analysis showed that novel miRNAs had a similar tendency in all libraries. The majority of these novel miRNAs had a uridine residue at the first position of the 5′ terminal and they were 21 nt long, except for those in YL, which had a length of 22 nt (Figure S1). The average negative minimal folding free energy of these miRNA precursors ranged from −171 to −19.90 kcal mol^−1^ with a mean of −58.10 kcal mol^−1^. These values were much lower than the minimal folding free energy of tRNA (−27.50 kcal mol^−1^) and rRNA (−33 kcal mol^−1^) (Bonnet et al., 2004).

The abundance of miRNAs was significantly different among the identified novel miRNAs. The expression levels of most of the novel miRNAs were low, generating fewer than 500 reads—21 (<100 normalized reads) miRNAs were found at low copies, only 13 (>1000 normalized reads) miRNAs were highly expressed in both leaf and fruit tissues—thereby indicating lower expression levels than those observed for conserved miRNAs. The complementary miRNA-star sequences (miRNA^*^) for each novel candidate miRNA were also detected. Of 115 novel miRNAs, 70 complementary sequences were found in the eight libraries. Most miRNA^*^ showed weak expression, and their expression levels were much lower than the expression of their corresponding miRNAs. This is consistent with the idea that miRNA^*^ strands are degraded rapidly during the biogenesis of mature miRNAs (Rajagopalan et al., 2006; Li et al., 2013). Intriguingly, Eu-miR8^*^ reads were extremely enriched in mature fruits, suggesting that the retention of star strands can occur in a tissue- and stage-specific manner. Eu-miR37^*^ were found to accumulate at the levels comparable to those of their respective miRNAs, thus indicating a functional importance of this miRNA^*^.

### Differential expression of miRNAs in *E. ulmoides*

We performed a differential expression analysis between the leaf and fruit libraries to detect the miRNA expression. All tested samples were sequenced with two biological replicates and the value of the Pearson correlation coefficient *r* between the two biological replicates was 0.796–0.902. Those miRNAs from each of the two biological replicates with overlapping differential expression were used for subsequent analyses. miRNAs with *P*-values lower than 0.05 were considered significantly altered. The comparison between YF and MF, YL and ML, YL and YF, and ML and MF identified 14 conserved miRNAs and 49 novel miRNAs that were differentially expressed (Table 2). The members of differentially expressed miRNAs were different in each group and they were involved in stage- or tissue-specific expression. Among these differentially expressed miRNAs, the pairwise analysis of the miRNA abundance between libraries identified 46 (19 increased, 27 decreased), 6 (1 increased, 5 decreased), 19 (9 increased, 10 decreased), and 24 (12 increased, 12 decreased) significantly differentially expressed miRNAs in YF vs. MF, YL vs. ML, YL vs. YF, and ML vs. MF groups, respectively. There were no miRNAs common for the four groups, and 26 miRNAs were present only in the YF vs. MF group. Differentially expressed Eu-miR27, Eu-miR36, and Eu-miR126 were found only in the YL vs. YF group and miR172a, miR164a, Eu-miR4, Eu-miR33, and Eu-miR103 only in the ML vs. MF group. These results suggest that miRNAs play a specific role in the biosynthesis of rubber in *E. ulmoides*. In addition, the expression pattern of miRNAs also revealed a dynamically regulated expression in leaves and fruits at different developmental stages. Sixteen miRNAs were dynamically distributed in these four groups. For instance, the expression levels of Eu-miR8 were up-regulated in YF vs. YF but suppressed in YF vs. MF and ML vs. MF. These results suggest that the expression of miRNAs was significantly altered at different developmental stages, and therefore, it might play important roles in biosynthesis of Eu-rubber in specific tissues at specific growth and developmental stages. In addition, among these differentially expressed miRNAs, the expression patterns of 36 miRNAs (4 conserved and 32 novel) were inversely correlated with the accumulation rate of Eu-rubber, suggesting these miRNAs were involved in regulating the biosynthesis of Eu-rubber.

**Table 2:** **Differential expression of miRNA genes in YF vs. MF, YL vs. ML, YL vs. YF and ML vs. MF of *Eucommia ulmoides***.

**miRNA name**	**miRNA sequence**	**Log2 fold change**			
		**YF/MF**	**YL/ML**	**YL/YF**	**ML/MF**
miR156g	UGACAGAAGAUAGAGAGCAC	−1.273	−2.474^**^	−0.451	2.826^**^
miR159	UUUGGAUUGAAGGGAGCUCUA	−1.205	−0.651	0.139	−0.250
miR160a	UGCCUGGCUCCCUGUAUGCCA	2.519	−0.186	−1.739^**^	0.910
miR164a	UGGAGAAGCAGGGCACGUGCA	−0.915	0.595	−0.933	−2.620^**^
miR166a	UCGGACCAGGCUUCAUUCCCC	−1.672	−0.021	1.123	−0.299
miR167a	UGAAGCUGCCAGCAUGAUCUGG	−1.111	−0.412	1.350	0.940
miR168d	UCGCUUGGUGCAGGUCGGGAC	−1.100	−0.134	0.185	−0.657^**^
miR172a	AGAAUCUUGAUGAUGCUGCAG	−0.243	0.032	1.652	1.631^**^
miR172c	AGAAUCUUGAUGAUGCUGCAU	−0.047	−0.072	2.343^**^	2.887^**^
miR390b	AAGCUCAGGAGGGAUAGCGCC	1.904	0.927	−2.402^**^	−1.577
miR396a	UUCCACAGCUUUCUUGAACUG	−1.312	−0.931	−0.283	−0.389
miR396b	UUCCACAGCUUUCUUGAACUU	−0.895	−0.425	0.607	0.469
miR408	UGCACUGCCUCUUCCCUGGCU	−1.434	1.063	0.839	−1.633^**^
miR477a	ACUCUCCCUCAAGGGCUUCUG	−2.344	−2.169	−0.419	0.262
Eu-miR1	ACCUGGCUCUGAUACCAUGAUAAC	0.942^**^	0.005	−1.336	−0.397
Eu-miR101	AUUCUCCCUCAAGGGCUUCUA	−2.291^**^	−1.410	1.381	1.345^**^
Eu-miR103	AUAACAAAAAUGAAUAUGGACUAA	0.345	0.354	1.201	1.395^**^
Eu-miR108	CGGAUUUGUGCUUUGGCGCAC	1.097^**^	0.246	0.711	1.515
Eu-miR111	UUCCGCCUAGGCAGUAGUUUCU	0.20168	0.245	−2.191^**^	−2.456^**^
Eu-miR112	UCGCAGGAAAGAUGGCACUUG	−2.141^**^	−1.095	0.473	−0.240
Eu-miR113	GCUCACUUCUCUCUCUGUCAGC	2.375^**^	−0.270	−1.084	1.195
Eu-miR115	CACGGAUGGUUUGAGCAUGGGAGU	2.465^**^	0.938	−0.964	0.240
Eu-miR118	UUGGCCAAUGUUGUCUUUCCGA	−1.526^**^	−0.284	0.908	−0.081
Eu-miR119	UUCGGACUCAUCUUUCGGGAAG	−0.079	*V*0.064	−3.810^**^	−3.489^**^
Eu-miR12	GUUCAAUAAAGCUGUGGGAAG	1.357^**^	0.820	−0.803	−0.576
Eu-miR126	UGGUAUUUUCGUCAUUACUUU	0.579	0.051	−2.742^**^	−1.811
Eu-miR14	AAGGUCUAGAGUUCAACUCCUUU	3.173^**^	2.824^**^	1.079	0.989
Eu-miR144	AAUAUUUCCGUCGAAAAUCAAAUC	1.299^**^	0.377	0.447	1.453^**^
Eu-miR146	CGUGAUAUUGUUUUGGCUCAAC	2.831^**^	1.407	1.056	1.308
Eu-miR15	UCUCGGACCAGGCUUCAUUCC	−2.286^**^	−0.869	1.371	0.343
Eu-miR16	UCGAAUUGAUUGUAGUGCACCACA	0.645^*^	0.142	−1.104	−0.608
Eu-miR18	CACUCUCCUUCAAAGACUUCCA	−3.019^**^	−2.956^**^	−0.332	0.048
Eu-miR24	AUUCUCCCUCAAGGGCUUCUC	−2.030^**^	−2.438^**^	−1.514	−0.427
Eu-miR26	UCGGGACCGGAAUAAUGCACA	−1.631^**^	−0.653	1.178	0.545
Eu-miR27	ACUGUCGCAGGAGAGAUGAUAC	−0.468	1.551	3.153^**^	1.281
Eu-miR29	AUUCUCCCUCAAGGGCUUCUG	−0.203	−0.640	1.806^**^	2.412^**^
Eu-miR33	UUUUGUUGAUGGUCAUCUAAUC	0.830	−0.666	0.707	2.476^**^
Eu-miR36	GGGAUUGUAGUUCAAUUGG	0.635	1.637	2.002^**^	0.995
Eu-miR38	UUGCUACGAUAACAUUUGCUUU	1.074^**^	−1.132	−2.866^**^	−0.651
Eu-miR4	UCGGACCAGGCUUCAUUCCUC	−0.030	1.272	−0.251	−1.617^**^
Eu-miR41	UCCGCAGGAGAGAUGAUACCG	0.876	0.244	4.339^**^	5.700^**^
Eu-miR43	UGAUCGAUAAACCUCUGCAUC	−1.206^**^	−0.761	0.497	0.300
Eu-miR44	AAGGGUUGUAAUUUUCGAUGC	2.681^**^	1.175	−4.586^**^	−2.869^**^
Eu-miR45	AGUGCCGUCUUCUUUGUGACA	−2.547^**^	−2.038^**^	−0.735	−0.812
Eu-miR46	GGAAUGUUGUCUGGCCCGAGG	1.261^**^	1.106	−0.371	−0.553
Eu-miR49	UUUGCCAGAGGAGAUUUGCAC	−1.886^**^	−1.872	1.367	2.403^**^
Eu-miR50	AGACAAGCUCUUCCCUCUCAUG	0.768	1.562	4.422^**^	4.676^**^
Eu-miR54	UAUCUUCUUUUGUCAAAUAUGUGG	−0.585	0.276	−2.484^**^	−2.586^**^
Eu-miR55	AUCUUAAUUGUCAGAGCAACAACA	2.146^**^	0.834	−0.083	1.214
Eu-miR56	CACGUGCUCCCCUUCUCCAAC	2.691^**^	1.206	−2.892^**^	−2.230^**^
Eu-miR59	CGAAUCCUGAUGAUGCUGCAU	−2.126^**^	−0.696	−1.045	−2.025^**^
Eu-miR61	AGGUCAUGUGGUAGCUUCACC	1.895^*^	1.723	−1.156	−1.088
Eu-miR66	ACCUGGUUCUGAUACCAUGAUAAC	1.366^**^	0.121	−1.542	−0.344
Eu-miR68	AACGGUUGCACUUAAGUCUUAAGC	1.841^*^	0.702	−0.063	1.026
Eu-miR69	ACACUUUACCCCUGUAGUUUG	−2.163^*^	−0.858	0.2849	−1.089
Eu-miR7	UGAAGCUGCCAGCAUGAUCUCA	−2.020^**^	−0.721	2.379^**^	1.496^**^
Eu-miR70	GCUCACUUCUCUUUCUGUCAGA	−2.072^**^	−0.585	3.385^**^	0.735
Eu-miR74	CAUCUGCUGGAUUAUGGC	−3.483^**^	−1.730	0.314	−0.742
Eu-miR77	GCUCUCUCUUCUUCUGUCACCA	−3.419^**^	−0.833	−0.721	−1.298
Eu-miR78	AGAAUGUCAAGUGAUCCAAGGACU	0.485	0.659	−1.804^**^	−2.266^**^
Eu-miR8	UUGUGUUCUCAGGUCACCCCU	−6.615^**^	0.444	1.766^**^	−5.378^**^
Eu-miR82	AUAUAGGGUAAGGCUGCGUAC	−2.698^**^	−2.294^**^	0.340	0.488
Eu-miR88	AAAUUGGCUAAACCACAGGGGGUA	−2.069^**^	−0.917	0.092	−0.628

The miRNA abundance was evaluated and normalized using the transcript per million (TPM) method, and the fold changes were computed from log_2_(YF/MF), log_2_(YL/ML), log_2_(YL/YF), and log_2_(ML/MF) based on their expression levels. Chi-squared tests were also performed to test the significance of each comparison, using the R software. p <0.01 indicates extreme significance (**), while p > 0.01 and p <0.05 are defined significant (*). The yellow indicates the pattern of differentially expressed miRNAs that were inversely correlated with the accumulation rate of Eu-rubber. YL, young leaves; ML, mature leaves; YF, young fruits; MF, mature fruits.

### Prediction and annotation of potential target genes

To elucidate the functions of conserved and novel miRNAs of *E. ulmoides*, we predicted putative targets by using web-based psRNATarget program with default settings. A total of 202 and 306 target mRNAs were found for 33 conserved and 92 novel miRNAs, respectively (Table S6). Seventy-two (14.17%) target gene sequences exhibited no functional annotation owing to the low expression. The annotated 436 predicted target genes belonged to a large number of transcription factors and functional gene families with different biological functions. A single miRNA were predicted to regulate several target genes, which usually belong to a large gene family. In our study, the number of target genes for each differential miRNAs ranged from 1 to 16, and the highest number of target genes was predicted for miR397, whereas 22 miRNAs (3 conserved miRNAs and 19 novel miRNAs) had only one target gene. In addition, the majority of miRNAs from the same family and even some miRNAs from different families could regulate the same target genes; for example, miR160 and miR159 could regulate the expression of auxin response factors.

The target genes of conserved miRNAs are involved in various biological processes including transcription factor, transcriptional regulation, signal transduction, and stress responses. A total of 65 putative transcription factors that are distributed in 17 families, which are involved in the regulation of gene expression and signal transduction, were identified (Table 3). For example, homeobox-leucine zipper protein (HD-ZIP) transcription factors, which are the largest group of transcription factors in the present study, were found to serve as the main target genes of miR166a; squamosa promoter-binding protein-like (SPL) transcription factors were predicted to act as target genes of miR156; NAC and MYB transcription factors were shown to be regulated by miR164a and miR159, respectively; auxin response factors (ARFs) played an important role in adaptive responses in plant growth and development and were predicted to be modulated by miR160; and miR172a, miR172j, and miR477b were the target for basic helix-loop-helix (bHLH) transcription factors. Many functional genes involved in disease resistance were predicted to be the target genes of the differentially expressed miRNAs; for instance, G-type lectin S-receptor-like serine/threonine-protein kinase was predicted to be a target of miR172a/172c/172j; miR397 was predicted to be a target of laccase protein; and the predicted target genes of miR167a/164c/167d and miR390a were involved in leucine-rich repeat receptor-like serine/threonine protein kinase. Although the novel miRNAs were sequenced at relatively lower levels than the conserved miRNAs, 92 out of 115 novel miRNAs were found to have candidate targets. Similarly, the target genes of the novel miRNAs also consisted of transcription factors and functional genes. For example, teosinte branched1/cycloidea/proliferating cell factor1 transcription factors were found to serve as the target genes of Eu-miR17, whereas Eu-miR118 and Eu-miR121 are the targets of disease resistance proteins.

**Table 3:** **Identified candidate transcription factor targets of miRNAs in *Eucommia ulmoides***.

**miRNA**	**Target gene**	**Classification**	**Annotation**
miR166a	EUC13752-RA	HD-ZIP	Homeobox-leucine zipper protein
miR166a	EUC16387-RA	HD-ZIP	Homeobox-leucine zipper protein
miR172j	EUC19561-RA	HD-ZIP	Homeobox-leucine zipper protein
Eu-miR101	EUC19481-RA	HD-ZIP	Homeobox-leucine zipper protein
Eu-miR126	EUC09594-RA	HD-ZIP	Homeobox-leucine zipper protein
Eu-miR15	EUC25692-RA	HD-ZIP	Homeobox-leucine zipper protein
Eu-miR4	EUC25115-RA	HD-ZIP	Homeobox-leucine zipper protein
Eu-miR55	EUC01299-RA	HD-ZIP	Homeobox-leucine zipper protein
Eu-miR83	EUC18098-RA	HD-ZIP	Homeobox-leucine zipper protein
miR156a	EUC01176-RA	SPL	Squamosa promoter-binding-like protein
miR156a	EUC06405-RA	SPL	Squamosa promoter-binding-like protein 12
miR156a	EUC15061-RA	SPL	Squamosa promoter-binding-like protein 16
miR156a	EUC17050-RA	SPL	Squamosa promoter-binding-like protein 6
miR156a	EUC17426-RA	SPL	Squamosa promoter-binding-like protein 14
miR156a	EUC19002-RA	SPL	Squamosa promoter-binding-like protein 6
miR156a	EUC25238-RA	SPL	Squamosa promoter-binding-like protein 13B
miR171b	EUC03199-RA	GRAS	Scarecrow-like protein 6
miR171b	EUC07008-RA	GRAS	Scarecrow-like protein 6
miR171c	EUC03236-RA	GRAS	Scarecrow-like protein 15
miR477a	EUC23508-RA	GRAS	DELLA protein RGL1
Eu-miR93	EUC16773-RA	GRAS	Scarecrow-like protein 9
Eu-miR93	EUC16774-RA	GRAS	Scarecrow-like protein 9
miR159	EUC04226-RA	ABI3VP1	ABI3VP1 transcription factor
miR164a	EUC01574-RA	AP2-EREBP	Ethylene-responsive transcription factor-like protein
miR172a	EUC00521-RA	AP2-EREBP	Ethylene-responsive transcription factor RAP2-7
miR172a	EUC17488-RA	AP2-EREBP	Floral homeotic protein APETALA 2
miR172a	EUC20986-RA	AP2-EREBP	Floral homeotic protein APETALA 2
miR172a	EUC21154-RA	AP2-EREBP	Ethylene-responsive transcription factor RAP2-7
miR164a	EUC00071-RA	NAC	NAC domain-containing protein 100
miR164a	EUC02290-RA	NAC	NAC domain-containing protein 21/22
miR164a	EUC03914-RA	NAC	NAC domain-containing protein 100
miR164a	EUC06685-RA	NAC	NAC domain-containing protein 100
miR164a	EUC14607-RA	NAC	NAC domain-containing protein 100
Eu-miR13	EUC11028-RA	NAC	Putative NAC domain-containing protein 94
miR160a	EUC13902-RA	ARF	Auxin response factor 17
miR160a	EUC16391-RA	ARF	Auxin response factor 18
miR160a	EUC16392-RA	ARF	Auxin response factor 18
miR160a	EUC19232-RA	ARF	Auxin response factor 18
Eu-miR30	EUC11854-RA	ARF	Auxin response factor 18
miR396a	EUC01918-RA	GRF	Growth-regulating factor 1
miR396a	EUC02016-RA	GRF	Growth-regulating factor 2
miR396a	EUC03434-RA	GRF	Growth-regulating factor 3
miR396a	EUC10096-RA	GRF	Growth-regulating factor 3
miR396a	EUC22328-RA	GRF	Growth-regulating factor 5
Eu-miR104	EUC06461-RA	TCP	Transcription factor TCP3
Eu-miR104	EUC20760-RA	TCP	Transcription factor TCP10
Eu-miR17	EUC07713-RA	TCP	Transcription factor TCP4
Eu-miR17	EUC13147-RA	TCP	Transcription factor TCP2
Eu-miR65	EUC22153-RA	TCP	Transcription factor TCP13
miR172a	EUC16991-RA	bHLH	Transcription factor bHLH78
miR477b	EUC18920-RA	bHLH	Putative transcription factor bHLH041
miR477b	EUC18922-RA	bHLH	Putative transcription factor bHLH041
Eu-miR97	EUC21432-RA	bHLH	ranscription factor bHLH137
Eu-miR13	EUC00701-RA	mTERF	DELLA protein RGL1
miR159	EUC16661-RA	MYB	Myb-related protein Myb4
Eu-miR17	EUC12082-RA	MYB	Transcription factor GAMYB
Eu-miR30	EUC15385-RA	MYB	Transcription factor GAMYB
Eu-miR47	EUC06624-RA	MYB	MYBC_MAIZE Anthocyanin regulatory C1 protein
miR319a	EUC00686-RA	C3H	Zinc finger CCCH domain-containing protein 6
Eu-miR2	EUC05291-RA	C3H	Zinc finger CCCH domain-containing protein 40
Eu-miR70	EUC12334-RA	EIL	Protein ETHYLENE INSENSITIVE 3
miR397	EUC08088-RA	MADS	Agamous-like MADS-box protein AGL9 homolog
miR396a	EUC06267-RA	mTERF	Mitochondrial transcription termination factor
Eu-miR89	EUC05832-RA	Trihelix	Trihelix transcription factor
Eu-miR89	EUC01591-RA	WRKY	Probable WRKY transcription factor 42

**Figure 3:**
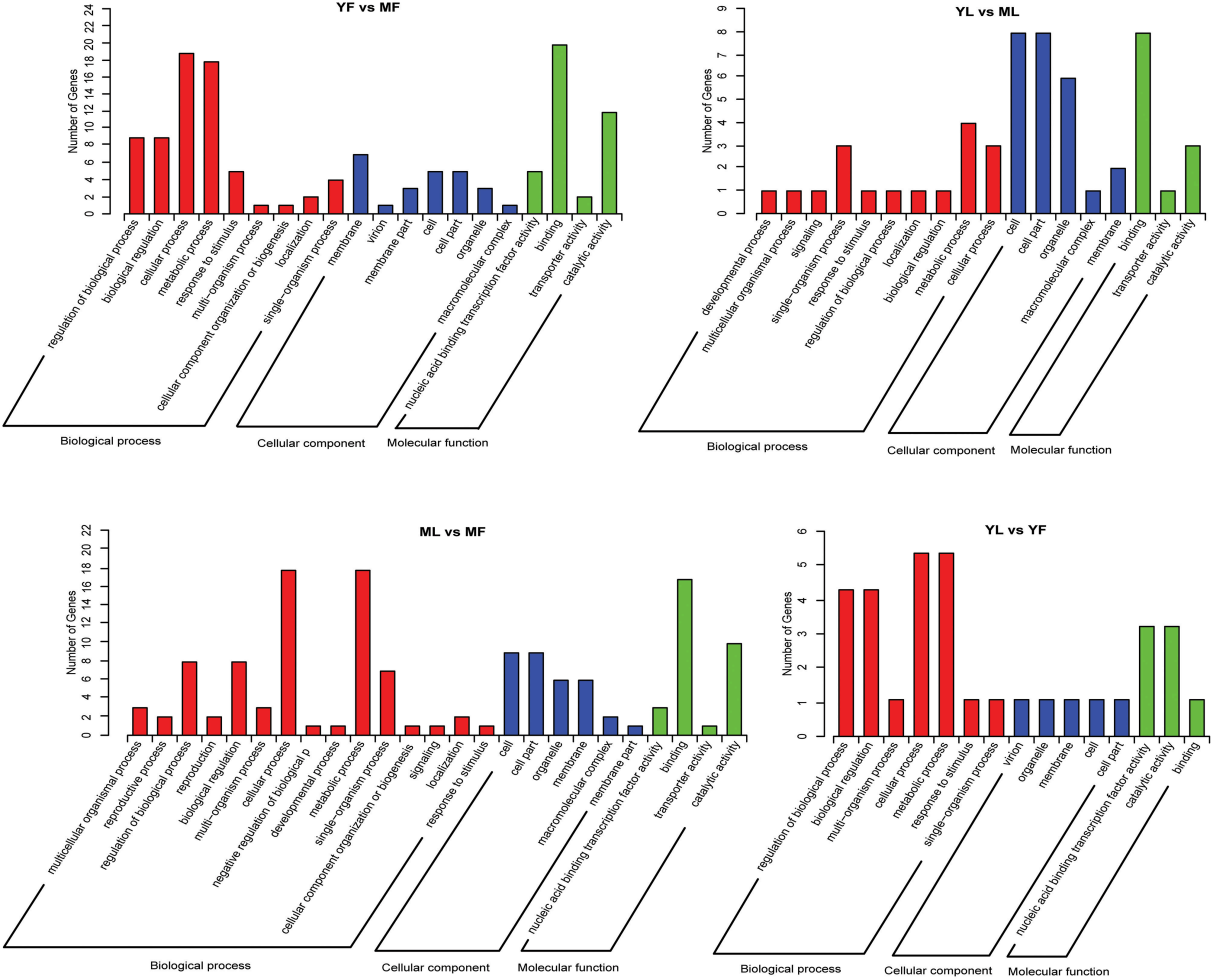
**Gene ontology (GO) enrichment analysis of target genes for the differentially expressed miRNAs in *Eucommia ulmoides***.

To gain insights into possible roles of differentially expressed miRNAs, putative target genes of these differentially expressed miRNAs were identified based on GO analysis and KEGG pathway analysis. GO annotation enrichment showed that the target genes could be summarized into three main categories in each group: biological processes, cellular components, and molecular functions (Figure 3). The majority of target genes involved in major biological processes were enriched in cellular process and metabolic process. In the cellular component group, the target genes were associated with cell, cell part, organelle, and membrane. Based on the molecular function category, the target genes were associated with binding and catalytic activity. The differential expression of target genes involved in various biological processes indicated that these target genes are responsible for biosynthesis of Eu-rubber from different tissues and at different developmental stages. Overall, a total of 24, 7, 23, and 20 different pathways enriched with target genes of differentially expressed miRNAs were found in the YF vs. MF, YL vs. ML, YL vs. YF, and ML vs. MF groups, respectively. The term metabolic pathways was the most dominant, followed by the biosynthesis of secondary metabolites (Table S7).

**Figure 4:**
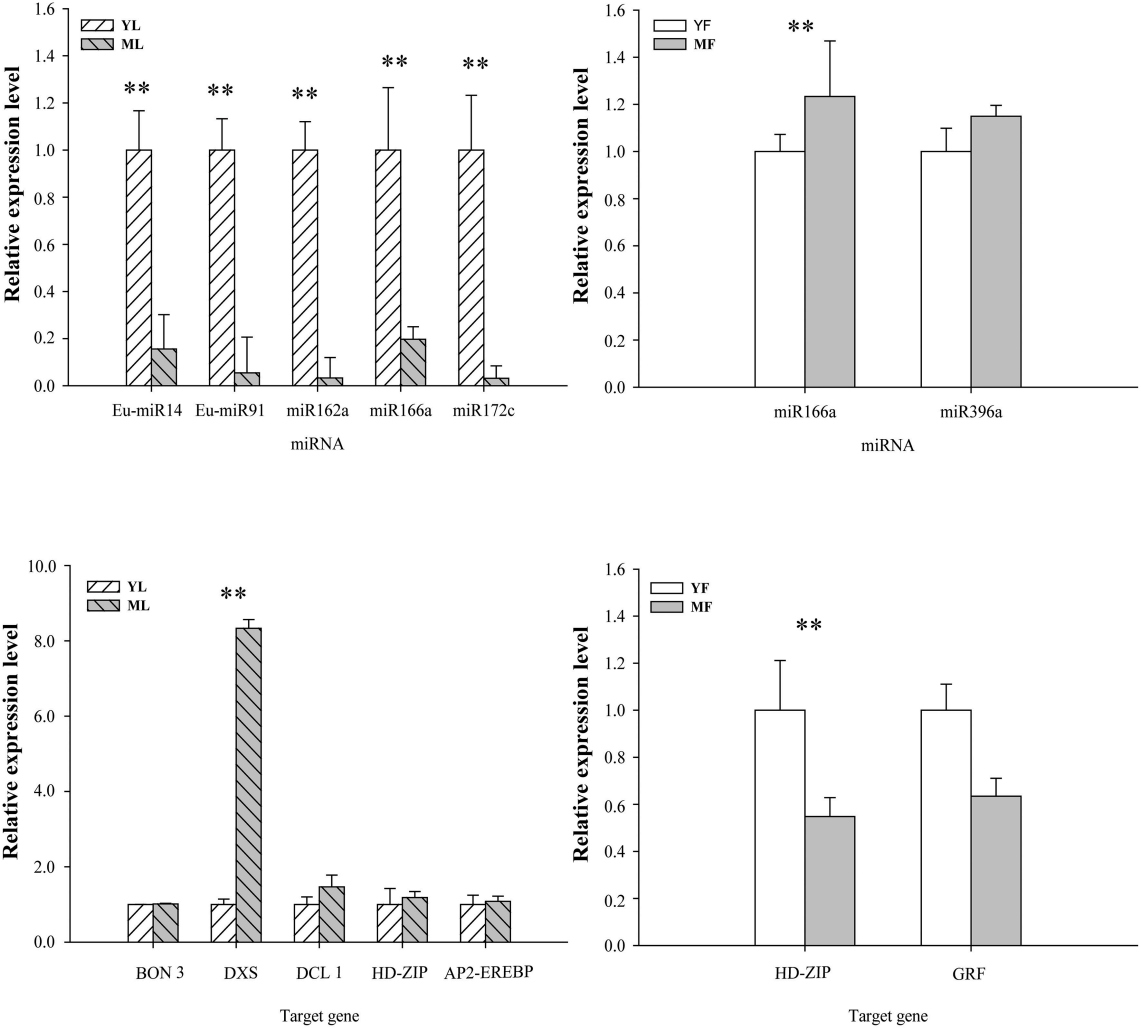
**qPCR analysis expression levels of miRNAs and their targets in *Eucommia ulmoides***. All data were subjected to analysis of variance (ANOVA) and the data represent the mean ± S.D. The results were considered statistically significant at (*p <0.05) or (**p <0.01).

To further evaluate the relationship between miRNAs and their target genes and confirm candidate genes involved in rubber accumulation, we performed qPCR to analyze the miRNAs and their targets, which may participate in rubber accumulation of *E. ulmoides* according to our sequencing data. The qPCR results for all tested miRNAs were consistent with the sequencing data and suggested a negative correlation between miRNAs expression and their putative target genes (Figure 4). Five miRNAs (Eu-miR14, Eu-miR91, miR162a, miR166a, and miR172c) were significantly upregulated in young leaves, whereas their target genes were downregulated. Moreover, miR166a and miR396a were negatively correlated with their putative targets in the YF vs. MF group. Eu-rubber is a secondary metabolite whose biosynthesis might be controlled by these target genes. The putative target gene of Eu-miR91 was predicted to be 1-deoxy-D-xylulose-5-phosphate synthase (DXS), the first rate-limiting enzyme in the MEP pathway, which was associated with the biosynthesis of terpenoids. The predicted target genes of miR166a, miR172c, and miR396a encoded transcription factors HD-ZIP, AP2-EREBP, and GRF. Moreover, Eu-miR14 and miR162a were predicted to target the genes of the protein bonzai 3 (BON 3) and the endoribonuclease dicer homolog 1 (DCL 1), respectively.

## Discussion

Eu-rubber is an important industrial gum found in the leaves, fruits, roots, and bark of *E. ulmoides*. Previous studies mainly centered on pharmacological applications, genetic diversity, and transcriptional levels of this species (Wang et al., 2006; Chen et al., 2012; Suzuki et al., 2012; Huang et al., 2013; Feng et al., 2015). In the present study, we identified miRNAs and associated target genes involved in Eu-rubber biosynthesis and thus provide valuable information about molecular regulatory mechanisms of Eu-rubber biosynthesis. Thus, eight sRNA libraries from young and mature leaves and fruits of *E. ulmoides* were constructed and sequenced. These libraries generated more than 95 million clean reads; of those, 60% were perfectly matched to the *E. ulmoides* genome, indicating the overall good quality of the sequence data. The 24 nt class exhibited the highest abundance, which was consistent with the distribution patterns of sRNAs reported in *Nicotiana benthamiana* (Baksa et al., 2015), *Panax notoginseng* (Wei et al., 2015), *Medicago sativa* (Fan et al., 2014), *Citrus trifoliata* (Song et al., 2010), *Taxus chinensis* (Qiu et al., 2009), and *Asparagus officinalis* (Chen et al., 2016). A total of 34 conserved miRNAs and 115 novel miRNAs were identified. Most of the identified miRNAs are highly conserved in most land plants, which suggests that they are deeply conserved in the plant kingdom and have a possible important role in plant growth and development (Cuperus et al., 2011).

Analyzing the temporal and spatial expression patterns of miRNAs would provide useful information about their molecular functions. The miRNAs previously identified in opium poppy, *Ricinus communis, Jatropha curcas, Medicago sativa*, and *Panax notoginseng* exhibited tissue- and stage-specific expression patterns, and therefore, play an important role in development and stress adaptation (Unver et al., 2010; Sun, 2012; Wang C. M. et al., 2012; Xu et al., 2013; Fan et al., 2014; Wei et al., 2015). The analysis of the conserved miRNAs showed that MIR159, MIR166, and MIR396 were highly expressed in both leaf and fruit tissues, which was consistent with the expression patterns in tobacco (Baksa et al., 2015). In this study, MIR159 (miR159/319) was the most abundant miRNA, accounting for more than 50% of the total sequence reads, thereby suggesting that MIR159 may have specialized functions in Eu-rubber biosynthesis. MIR159 targets four MYB proteins, which contain a conserved MYB DNA binding domain and are one of the larger group of plant protein family that play a regulatory role in developmental processes and defense responses (Wu and Poethig, 2006; Fornara and Coupland, 2009). In *Arabidopsis*, MYB transcription factors are involved in plant leaf development (Palatnik et al., 2003; Millar and Gubler, 2005), whereas in *Hevea brasiliensis*, a MYB transcription factor induces programmed cell death in rubber tree bark, and its expression is significantly decreased in the latex of trees affected by tapping panel dryness (Gébelin et al., 2013). MIR396 was predicted to target 17 growth-regulating factor genes encoding respective putative transcription factors, which play a regulatory role in plant growth and development as well as in defense responses (Wu and Poethig, 2006). In *E. ulmoides*, miR396 is expressed in high levels in mature tissues; this pattern of miR396 accumulation is in accord with that observed in *Arabidopsis* and *H. brasiliensis*, where it is exclusively expressed in mature leaves (Wang et al., 2011; Lertpanyasampatha et al., 2012). In addition, different conserved miRNAs, even those in the same family, exhibited different expression levels in various tested tissues, suggesting their specific potential roles in growth and development of *E. ulmoides*. Most of the newly identified miRNAs showed lower abundance levels than the conserved miRNAs, which suggested that these novel species-specific miRNAs are either young miRNAs that arose recently through evolution or they are more volatile than the other miRNAs (Jones-Rhoades et al., 2006; Rajagopalan et al., 2006; Song et al., 2010; Cuperus et al., 2011; Mao et al., 2012; Chen et al., 2016).

To better understand the functions of miRNAs, it is necessary to predict and annotate their target genes. The prediction in this study showed that these target genes have a wide range of functions, with the majority of the targets being transcription factors related to plant growth and development, including HD-ZIP, SPL, NAC, MYB, ARFs, bHLH, and WRKY transcription factors. Previous research indicated that miRNA families in different plants are highly conserved, and perform analogous regulatory functions (Zhao et al., 2010). miR166 was predicted to regulate HD-ZIP transcription factors, which are unique to plant kingdom; they are involved in organ morphogenesis and regulation of meristem growth in *Arabidopsis thaliana* (Green et al., 2005; Wang H. et al., 2013). miR164 regulates NAC-domain gene necessary for normal embryonic, vegetative, and floral development in *Arabidopsis* (Mallory et al., 2004). bHLH3 transcription factor, a target gene of miR828, regulates the accumulation of anthocyanins in apple after debagging (Qu et al., 2016). miR156 is highly conserved in plants and predicted to target the SPL transcription factor, which plays an important part in the spatiotemporal regulation of sesquiterpene biosynthesis in *A. thaliana* and *Pogostemon cablin* (Yu et al., 2015). miR171 targets mRNAs coding for the scarecrow-like proteins, which is a transcription factor that has been implicated in gibberellins synthesis in *Arabidopsis* (Llave et al., 2002b). WRKY transcription factors have been reported to play roles in regulating the biosynthesis of terpenoids in various plants including *Panax quinquefolius* and *P. notoginseng* (Eulgem et al., 2000; Ma et al., 2009; Wang J. et al., 2012; Yang et al., 2012; Wei et al., 2015). In our study, miR166a, miR156, miR164a, miR159, miR160, miR172, and Eu-miRn89 were predicted to target HD-ZIP, SPL, NAC, MYB, ARFs, bHLH, and WRKY transcription factors, respectively. The conservation of putative target genes suggests that these transcription factors also share the same function in the growth and development of *E. ulmoides*, especially in the synthesis of secondary metabolites. Furthermore, several miRNA targets encode proteins involved in responses to environmental stresses, such as heat shock protein (HSP). HSP, which is induced by heat shock stress conditions and is critical in cellular homeostasis under adverse environmental conditions, is the target of miR397, Eu-miR1, and Eu-miR166.

The qPCR analysis was used to identify candidate miRNAs and their predicted target genes, which were associated with regulation genes involved in the accumulation of rubber in *E. ulmoides*. The expression patterns of five (Eu-miR14, Eu-miR91, miR162a, miR166a, and miR172c) and three (miR166a, miR396a, and miR396b) miRNAs, which were consistent with the accumulation of Eu-rubber in YL vs. ML and YF vs. MF, respectively, were selected. miRNAs usually negatively regulate the expression of mRNA, and the selected miRNAs expression patterns exhibited inverse correlation with their corresponding targets, suggesting that miRNAs regulate the accumulation of Eu-rubber by post-transcriptionally regulating target genes to enhance rubber content in *E. ulmoides*. In plants, terpenoid is synthesized from isopentenyl diphosphate and its allylic isomer dimethylallyl diphosphate via two different pathways, the MVA pathway in the cytosol and the MEP pathway in cellular plastids, where the *DXS* gene catalyzes the first rate-limiting enzyme for isopentenyl diphosphate synthesis in the MEP pathway (Pulido et al., 2012). And previous reports have suggested that the MEP pathway is also involved in rubber biosynthesis in *E. ulmoides* and *Hevea brasiliensis* (Ko et al., 2003; Chow et al., 2007; Bamba et al., 2010). Several miRNAs were computationally predicted to directly target known genes in the terpenoid backbone biosynthesis pathway, including *DXS* (Wei et al., 2015). Therefore, in the present work, *DXS* was predicted to be a target of Eu-miR91, the expression levels of Eu-miR91 in young leaves was higher than that in mature leaves, and the expression of the target gene *DXS* was a perfect inverse to that of Eu-miR91, which suggest that reduced expression of Eu-miR91 could promote the accumulation of rubber by *DXS* gene. The negative correlation between the expression of miR166a, miR172c, and miR396a and their target genes encoding transcription factors were also revealed. These miRNAs may regulate Eu-rubber accumulation via suppression of their target gene expression. Previous studies have showed that *Arabidopsis thaliana* homeobox 12 (ATHB12), a homeodomain-leucine zipper class I (HDZip I) gene, can regulatethe cell growth during leaf development (Hur et al., 2015). Our results indicated that the HD-ZIP expression enhanced rubber content by promoting leaf and fruit growth. The negative correlation between the expression of miR166a and its target genes encoding HD-ZIP transcription factor was revealed, therefore implying that miR166a participate in the process of rubber biosynthesis in *E. ulmoides* via regulating the HD-ZIP gene. AP2-EREBP is the majority of stress responsive genes involved in ethylene responses, and has been characterized that positively regulates rubber biosynthesis in *Hevea brasiliensis* (Li et al., 2016). In our results, the expression of the target gene AP2-EREBP was a perfect inverse to that of miR172c—the expression of miR172c was down regulated in mature leaves, whereas the expression of AP2-EREBP was up regulated, suggesting that miR172c promotes the accumulation of rubber via AP2-EREBP in *E. ulmoides*. miR396 is conserved among dicot and monocot plants, and it targets the GRF genes encoding putative transcription factors involved in growth and development. A previous study showed that miR396 is abundant in mature tissues (Wang et al., 2011; Lertpanyasampatha et al., 2012), which was corroborated by the lower expression of miR396a in young fruits compared to that in in mature fruits observed in the present study. Moreover, the expression of miR396a was negatively correlated to GRF transcription factor, implying that miR396 might be negatively correlated with the accumulation of Eu-rubber. Our study is the first to associate these three transcription factors, HD-ZIP, AP2-EREBP, and GRF, with the accumulation of rubber, and thus, these transcription factors are candidate genes that regulate Eu-rubber accumulation in *E. ulmoides*.

Eu-rubber accumulation is a complex process involving many precursors and a series of regulatory genes. Thus, it is simplistic to assume a simple correlation between miRNA expression and their targets when identifying the mechanism of Eu-rubber accumulation, and further characterization of these miRNAs is required.

## Conclusions

In conclusion, the present study is the first genome-wide investigation of miRNAs and their targets in *E. ulmoides*. Eight sRNA libraries were generated and sequenced from *E. ulmoides* leaves and fruits at both young and mature stages using high-throughput sequencing technology. We identified 34 conserved miRNAs and 115 novel miRNAs and characterized their expression patterns, target genes, and function. Furthermore, six miRNAs and their targets that were involved in the accumulation of Eu-rubber were revealed. Although the foundation of the complex miRNA-mediated regulatory networks remains to be resolved, this miRNA dataset will help to elucidate the gene regulatory networks in *E. ulmoides* and other species. Therefore, this study provides a valuable source for studying complex gene regulatory functions of miRNAs, especially in the rubber accumulation of *E. ulmoides*.

## Author contributions

LW performed the experiments, data analysis and drafted the manuscript. HD and TW helped to conceive the study and participated in the design and coordination. All authors read and approved the final manuscript.

### Conflict of interest statement

The authors declare that the research was conducted in the absence of any commercial or financial relationships that could be construed as a potential conflict of interest.

## Supplementary material

Table S1 **Primers used in qPCR analysis of miRNAs and target genes**.

Table S2 **Quality evaluation of sequencing data**.

Table S3 **Number of reads for each classified small RNA identified in *Eucommia ulmoides***.

Table S4 **Conserved miRNAs identified from *Eucommia ulmoides***. Expression profiles of conserved miRNAs were estimated by transcript per million (TPM). Heat map colors represent absolute normalized levels of miRNA expression ranging from less than 10 RPM to more than 1000 RPM, as indicated in the color key.

Table S5 **Predicted novel miRNAs and their pre-miRNA sequences in *Eucommia ulmoides***. Expression profiles of conserved miRNAs were estimated by transcript per million (TPM). Heat map colors represent absolute normalized levels of miRNA expression ranging from less than 10 RPM to more than 1000 RPM, as indicated in the color key.

Table S6 **Target prediction of miRNAs in *Eucommia ulmoides***.

Table S7 **KEGG pathway analysis of genes for differentially expressed miRNAs in *Eucommia ulmoides***.

Figure S1 **First nucleotide bias of novel miRNA in *Eucommia ulmoides* young leaves (A)**, mature leaves **(B)**, young fruits **(C)**, and mature fruits **(D)**.

## Availability of supporting data

The small RNA sequencing raw data were deposited in the National Center for Biotechnology Information Sequence Read Archive (NCBI SRA) under the accession number SRP074871.

## Abbreviations

AP2-EREBP: ethylene-responsive transcription factor
ARFs: auxin response factors
bHLH: basic helix-loop-helix
BON 3: protein bonzai 3
DCL 1: endoribonuclease dicer homolog 1
DXS: 1-deoxy-D-xylulose-5-phosphate synthase
Eu-rubber: *Eucommia* rubber
GO: gene ontology
GRF: Growth-regulating factor
HD-ZIP: homeobox-leucine zipper protein
HSP: heat shock protein
KEGG: Kyoto Encyclopedia of Genes and Genomes
MEP: methylerythritol phosphate
miRNA: microRNA
miRNA*: miRNA-star sequences
MVA: mevalonate
SPL: squamosa promoter-binding protein-like
sRNA: small RNA
TPI, trans-1: 4-polyisoprene.
