# Supplementary figures and images for: Genome-Wide Identification of MicroRNAs and Their Targets in the Leaves and Fruits of Eucommia ulmoides Using High-Throughput Sequencing
Source: Front Plant Sci. 2016 Nov 8;7:1632. doi: 10.3389/fpls.2016.01632 (PMC5099690; doi:10.3389/fpls.2016.01632)

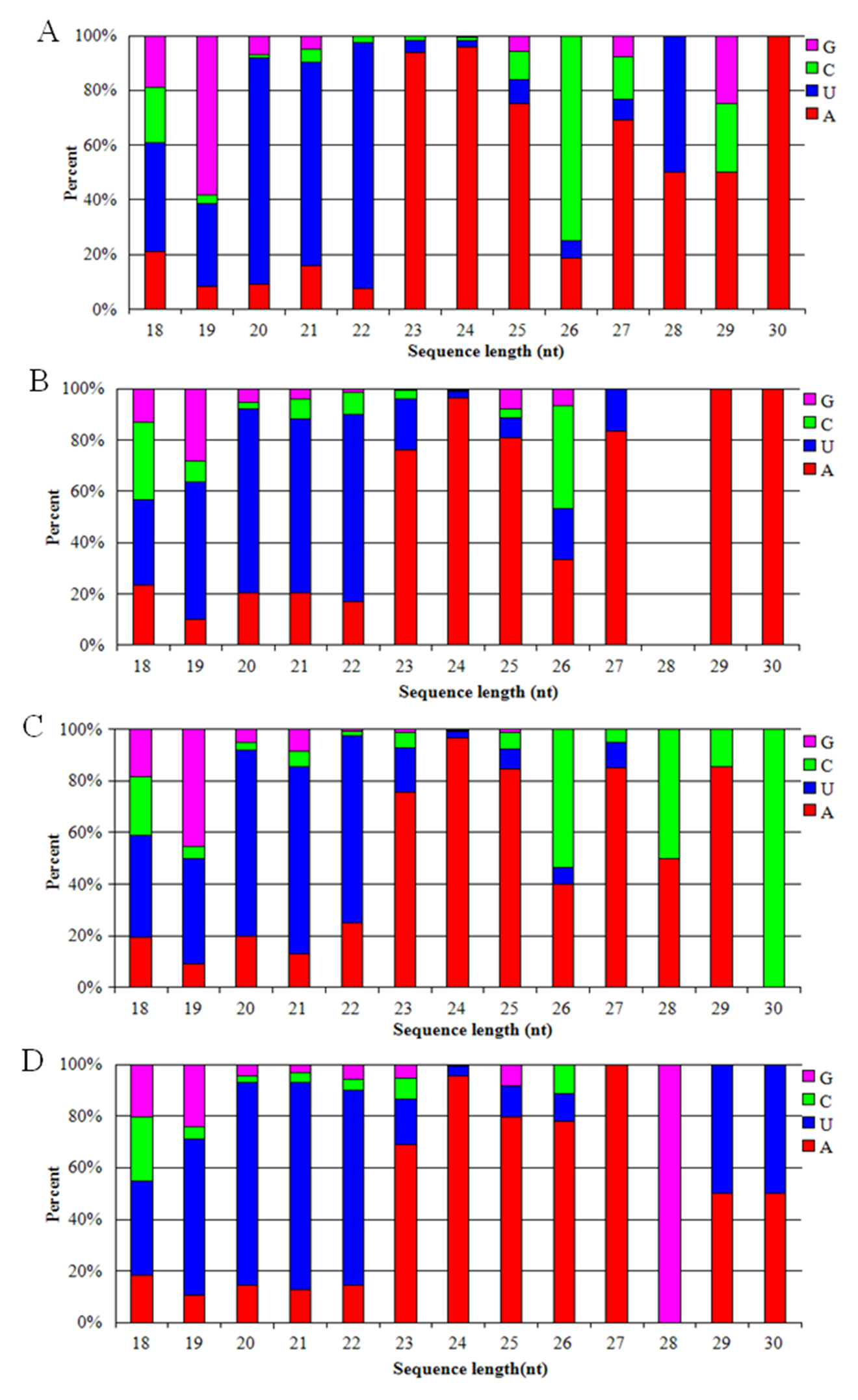

Supplement: Figure S1 — First nucleotide bias of novel miRNA in Eucommia ulmoides young leaves (A), mature leaves (B), young fruits (C), and mature fruits (D). [file Image1.TIF]
